# Electro-optical properties of strained monolayer boron phosphide

**DOI:** 10.1038/s41598-023-37099-9

**Published:** 2023-06-17

**Authors:** Mohammad Mortezaei Nobahari

**Affiliations:** Department of Physics, Ferdowsi University of Mashhad, Mashhad, Iran

**Keywords:** Condensed-matter physics, Optical physics

## Abstract

In this paper, we use tight-binding approximation and linear response theory to study the electronic and optical properties of strained monolayer boron-phosphide (h-BP). Compared with the previous DFT study and adding on-site energy variation to the Hamiltonian, we propose a theoretical approach to investigate the strain effects on the electronic and optical properties of the h-BP. Applying tensile strain increases the gap while compressive strain reduces it as the maximum and minimum of the gap are 1.45 eV and 1.14 eV respectively and are related to the biaxial strain. Also, we investigate the optical conductivity and electron energy loss spectrum (EELS) of the pristine and strained h-BP. The absorption peak of the $\Re\sigma_{xx,yy}$ appears in energy about 4 eV but applying strain shifts the peak’s energy. Optical properties of pristine h-BP are isotopic and biaxial strain preserves this isotropy, but uniaxial strain exerts anisotropic behavior in the system.

## Introduction

Today, two-dimensional (2D) materials have attracted researchers due to their inherent features. Inspired by graphene, the researchers were able to synthesize other 2D materials, such as silicone^1^, phosphorene^2^, borophene^3^, etc. Today, a large number of 2D materials have been synthesized. Looking for novel alternative materials is still interesting and challenging for various practical applications requiring different finite band gaps and on/off ratios. Phosphorene is a good candidate with a moderate band gap, good carrier mobility, and a high on/off ratio to replace silicene and graphene in the nanoelectronics field but, phosphorene has a fatal weakness in structural stability.

Among the 2D materials, boron-phosphide has good structural and mechanical stability and high carrier mobility^4–6^. Unlike graphene, the h-BP is a semiconductor with a direct band gap of 0.9–1.37 eV^6–8^. A recent theoretical study shows that n-type and p-type doped boron-phosphide monolayer can be used as an ideal 2D p–n junction, exhibiting diode characteristics with high current rectification and negative differential resistance^5^. Using ab initio studies on phonon modes without imaginary frequencies and molecular dynamics simulations, it has been well-proven that h-BP is stable at high temperatures and atmospheres^4,9–11^. Experimentally, epitaxial growth of h-BP films was synthesized on aluminum nitride (AIN)/sapphir substrates using chemical vapor deposition^12^. Other boron-phosphide allotropes have been predicted by density functional theory (DFT) calculations^13^.

Recently, a new member of hexagonal and orthorhombic 2D materials has been predicted using first principles and DFT calculations^14–16^. These semiconductor materials structurally are similar to the h-BP, but due to their different atomic composition and electronic structure, they possess distinct electronic and optical properties. Furthermore, the methodology used in these articles differs from those studied in previous works. The effects of atomic structure, external electric field, and stacking orders on the electronic properties of few-layer boron-phosphide are studied theoretically^17–20^. In a study by Wei et al.^21^, they investigated the effect of vacancy defects on the electronic properties of the monolayer boron nitride and found that B-vacancy, N-vacancy, and double vacancy defects can eliminate bandgap from direct to indirect. Among various ways to modulate the electronic features of 2D systems, in general, defects and strains in the process of synthesis of 2D materials are ineluctable^21–27^, and effort to understand strain effects on the synthesized materials is very important in the nanoelectronic industry^28,29^.

There are many methods to apply strain to the 2D materials in the experiment such as bending^30,31^, stretching^32^, pressure^33^, substrate-induced strain^34^, and thermal expansion^35^. The most common method for applying strain is to use a substrate with a different lattice constant. When the material is grown or transferred onto such a substrate, it experiences strain due to the mismatch in lattice constants, which can be controlled by adjusting the temperature and pressure during growth or transfer.

To date, several studies have been published about the strain effect on similar materials such as boron nitride, but they didn’t consider on-site energy modification by strain. In this study, we also added the on-site energy variation to the Hamiltonian and proposed a theoretical method to study the strain effect on such materials. Although many DFT studies have addressed the strain effect on the h-BP, there is no study that uses a tight-binding approximation to date. The tight-binding approximation is a powerful tool for studying the material’s properties. Due to the remarkable similarity of this approximation with DFT calculations^36–38^, we would also employ it to study the electro-optical properties of strained h-BP. The purpose of this paper is to study strain effects on the electronic and optical properties of the h-BP based on tight-binding approximation compared to other DFT methods. We also use linear response theory along with the Kubo formula to calculate the intra-band and inter-band optical conductivity (IOC) as well as EELS.

The paper is organized as follows. In Section “Theoretical background” we present the theoretical background for the electronic and optical properties of the pristine and strain-induced h-BP. The numerical results for the pristine and strained cases are discussed in Section “Discussion”. In Section “Conclusions” we will present a summary of the results.

## Theoretical background

### Pristine h-BP

**Figure 1:**
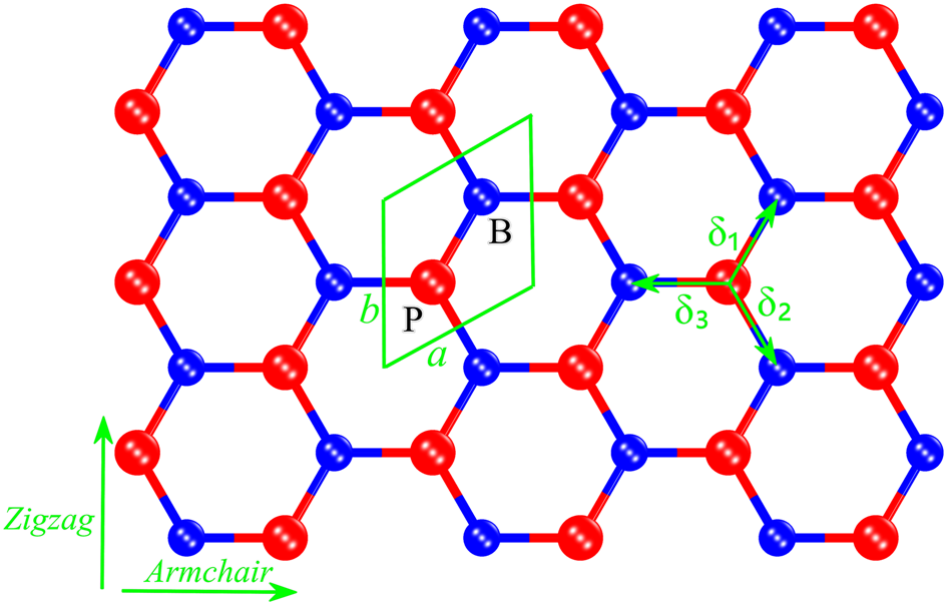
Geometry structure of the h-BP. The boron (B) and phosphorus (P) atoms are represented in blue and red colors respectively. The nearest-neighbor vectors are represented by ${\delta }_{j}$ (j = 1, 2, 3). The unit cell is shown as a parallelogram with length a = b = 3.213 Å and consists of two atoms. This figure is generated by CrystalMaker version 10.7.1.300.

**Figure 2:**
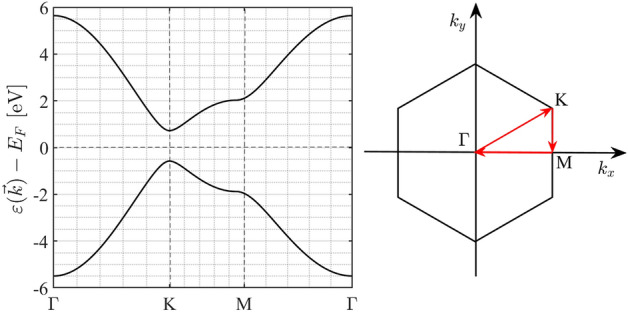
Band structure of the pristine h-BP in Γ-K-M-Γ direction. Right: first Brillouin zone of the monolayer h-BP.

**Figure 3:**
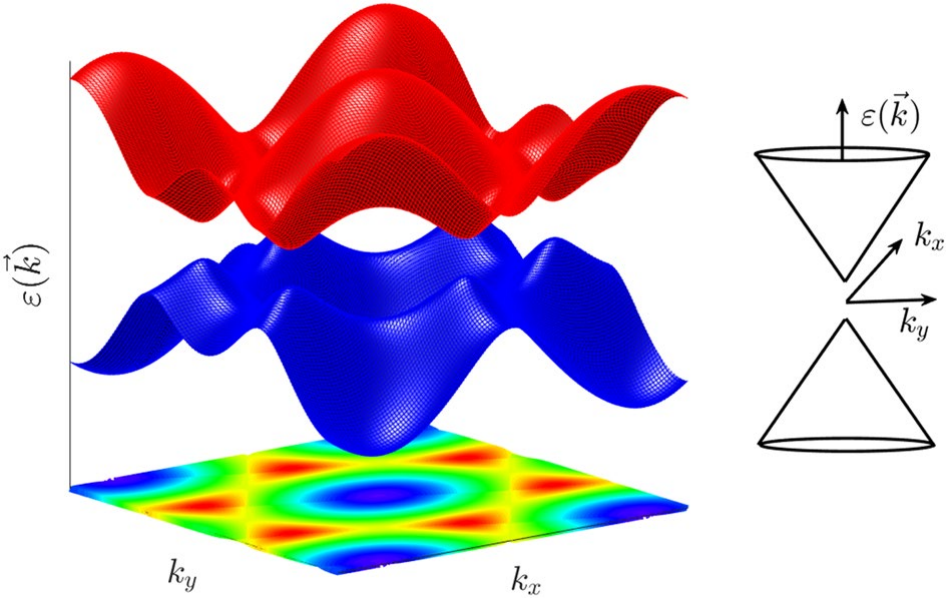
Band structure of the pristine h-BP with 3D first Brillouin zone. Right: Dirac cone around the K point. This figure is generated by Matlab version 9.4.0.813654 (R2018a).

h-BP, like graphene, has a honeycomb structure, but different lattice constant and on-site energies. Figure 1 shows the geometry structure of the h-BP with two different $B$ (boron) and $P$ (phosphorus) atoms that are displayed in blue and red colors respectively. The lattice constant of the h-BP is $a=b=3.211$ Å and $B-P$ bond length is ${a}_{0}=1.861$ Å^6^. Overlap and next-nearest-neighbor hopping have a low contribution in our calculation^39^, and we can ignore them. Therefore, the tight-binding Hamiltonian of the h-BP considering the nearest neighbors is

1 $$H=\left(\begin{array}{ll}{\varepsilon }_{p}& {t}_{0}\phi \\ {t}_{0}{\phi }^{*}& {\varepsilon }_{b}\end{array}\right)$$

where ${\varepsilon }_{p}=-3.276$ and ${\varepsilon }_{b}=-1.979$ eV^19^ are on-site energies of P and B atoms respectively, $\phi ={e}^{-i{k}_{x}a}+2{e}^{i{k}_{x}a/2}\mathrm{cos}\left(\sqrt{3}{k}_{y}a/2\right)$ is the structural factor and ${t}_{0}=-1.844$ eV is the hopping parameter. By diagonalization of Eq. (1) energy dispersion of the h-BP is

2 $${\varepsilon }_{\nu }\left(\overrightarrow{k}\right)=\frac{{\varepsilon }_{p}+{\varepsilon }_{b}}{2}+\nu \sqrt{{\left(\frac{{\varepsilon }_{p}-{\varepsilon }_{b}}{2}\right)}^{2}+{t}_{0}^{2}{\left|\phi \right|}^{2}}$$

where $\nu =\pm 1$ referred to the conduction and valence bands respectively. Figure 2 shows the h-BP band structure along the $\Gamma -K-M-\Gamma$ in first Brillouin zone (FBZ) with coordinates $\Gamma =\left(0,0\right)$, $K=\left(2\pi /3{a}_{0},2\pi /3\sqrt{3}{a}_{0}\right)$, and $M=\left(2\pi /3{a}_{0},0\right)$. As shown in Fig. 2 the minimum of the conduction band and the maximum of the valence band coincide at the $K$ point therefore by substituting these coordinates in Eq. (2) the h-BP gap is ${\varepsilon }_{g}=\left|{\varepsilon }_{p}-{\varepsilon }_{b}\right|=1.297$ eV. The Fermi energy locates at the midgap between the conduction and the valence bands in the energy equal to -2.627 eV. Figure 3 shows a 3D overview of the h-BP band structure. We can see the similarity between the graphene and h-BP band structures except in the gap amount.

### Hamiltonian of the strain-induced h-BP

In the pristine case, all hopping parameters are equal due to the equality of the nearest-neighbor lengths. But, when the strain is applied, the length of the nearest neighbors changes, and we have different hopping parameters. The hopping parameters ${t}_{j}$ are a function of the ${\delta }_{j}$ as ${t}_{1}\left({\delta }_{1}\right)$, ${t}_{2}\left({\delta }_{2}\right)$ and ${t}_{3}\left({\delta }_{3}\right)$, that ${\delta }_{j}$ are nearest-neighbor vectors as $\overrightarrow{{\delta }_{1}}={a}_{0}\left(1/2,\sqrt{3}/2\right)$, $\overrightarrow{{\delta }_{2}}={a}_{0}\left(1/2,-\sqrt{3}/2\right)$, and $\overrightarrow{{\delta }_{3}}={a}_{0}\left(-1,0\right)$ (see Fig. 1). The strain-induced h-BP Hamiltonian is

3 $$H=\left(\begin{array}{ll}\varepsilon {{^{\prime}}}_{p}&\quad \sum_{j=1}^{3}{t}_{j}{e}^{i\overrightarrow{k}.\overrightarrow{{\delta }_{j}}}\\ \sum_{j=1}^{3}{t}_{j}{e}^{-i\overrightarrow{k}.\overrightarrow{{\delta }_{j}}}&\quad \varepsilon {{^{\prime}}}_{b}\end{array}\right)$$

where ${t}_{j}$ is the modified hopping parameter by strain and $\varepsilon {{^{\prime}}}_{p}$ and $\varepsilon {{^{\prime}}}_{b}$ are on-site energies of the strained h-BP. The energy dispersion relation is

4 $${\varepsilon }_{\nu }\left(\overrightarrow{k}\right)=\frac{{\varepsilon }_{p}{^{\prime}}+{\varepsilon }_{b}{^{\prime}}}{2}+\nu \sqrt{{\left(\frac{{\varepsilon }_{p}{^{\prime}}-{\varepsilon }_{b}{^{\prime}}}{2}\right)}^{2}+|\sum_{j=1}^{3}{t}_{j}{e}^{i\overrightarrow{k}.\overrightarrow{{\delta }_{j}}}{|}^{2}}.$$

### Uniaxial strain effects on the nearest-neighbor bonds

In the real space, if $\overrightarrow{{\rho }_{0}}$ represents a pristine bond vector, the new bond length after stretching is equal to

5 $$\rho =\left(I+{\varvec{\upvarepsilon}}\right).\overrightarrow{{\rho }_{0}},$$

where $I$ is the unitary matrix. Since the h-BP hopping parameters depend on the nearest-neighbor lengths, we need to calculate the deformed bond lengths. Using Eq. (5) the magnitude of the nearest-neighbor lengths read as

6 $$\left|{\delta }_{1}{^{\prime}}\right|={a}_{0}\left(1+\frac{{\varepsilon }_{xx}}{4}+\frac{3{\varepsilon }_{yy}}{4}+\frac{\sqrt{3}{\varepsilon }_{xy}}{2}\right).$$

7 $$\left|{\delta }_{2}{^{\prime}}\right|={a}_{0}\left(1+\frac{{\varepsilon }_{xx}}{4}+\frac{3{\varepsilon }_{yy}}{4}-\frac{\sqrt{3}{\varepsilon }_{xy}}{2}\right).$$

8 $$\left|{\delta }_{3}{^{\prime}}\right|={a}_{0}\left(1+{\varepsilon }_{xx}\right).$$

where we have considered ${\varepsilon }_{xy}={\varepsilon }_{yx}$ and the second order of the strain is neglected.

Our preferred directions for applying strain are along the armchair(AC), zigzag (ZZ), and uniform biaxial strain. By considering our desired directions, strain tensors are as follows

9 $${{\varvec{\upvarepsilon}}}_{\mathbf{Z}}=\varepsilon \left(\begin{array}{cc}0& 0\\ 0& 1\end{array}\right),$$

10 $${{\varvec{\upvarepsilon}}}_{\mathbf{A}}=\varepsilon \left(\begin{array}{cc}1& 0\\ 0& 0\end{array}\right),$$

11 $${{\varvec{\upvarepsilon}}}_{\mathbf{B}}=\varepsilon \left(\begin{array}{cc}1& 0\\ 0& 1\end{array}\right),$$

where ${{\varvec{\upvarepsilon}}}_{\mathbf{A}}$, ${{\varvec{\upvarepsilon}}}_{\mathbf{Z}}$, and ${{\varvec{\upvarepsilon}}}_{\mathbf{B}}$ are the AC, ZZ, and biaxial strain tensors, and $\varepsilon$ is the applied strain. Therefore, the length of the new bonds after applying strain along the desired directions is given as

12 $$\begin{aligned}ZZ: \left|{\delta }_{1}{^{\prime}}\right|&= \left|{\delta }_{2}{^{\prime}}\right|={a}_{0}\left(1+\frac{3}{4}\varepsilon \right)\\ \left|{\delta }_{3}{^{\prime}}\right|&= {a}_{0}.\end{aligned}$$

13 $$\begin{aligned}Biaxial: \left|{\delta }_{1}{^{\prime}}\right|=\left|{\delta }_{2}{^{\prime}}\right|&= {a}_{0}\left(1+\frac{\varepsilon }{4}+\frac{3\varepsilon }{4}\right)\\ \left|{\delta }_{3}{^{\prime}}\right|&={a}_{0}\left(1+\varepsilon \right).\end{aligned}$$

14 $$\begin{aligned}AC: \left|{\delta }_{1}{^{\prime}}\right|&= \left|{\delta }_{2}{^{\prime}}\right|={a}_{0}\left(1+\frac{1}{4}\varepsilon \right)\\ \left|{\delta }_{3}{^{\prime}}\right|&= {a}_{0}\left(1+\varepsilon \right).\end{aligned}$$

As in real space, the strain also affects lattice vectors in reciprocal space and causes high symmetry points to shift. Using Eq. (5) the new position of the $M$ and $K$ points are given as

15 $$M{^{\prime}}=\frac{2\pi }{3{a}_{0}}\left(1-{\varepsilon }_{xx},-{\varepsilon }_{xy}\right).$$

16 $$K{^{\prime}}=\frac{2\pi }{3{a}_{0}}\left(1-{\varepsilon }_{xx}-\frac{{\varepsilon }_{xy}}{\sqrt{3}},-\frac{{\varepsilon }_{yy}}{\sqrt{3}}-{\varepsilon }_{xy}+\frac{1}{\sqrt{3}}\right).$$

### Hopping renormalization

One method to obtain the hopping energies as a function of the bond length is to use Harrison’s rule ($t\propto 1/{l}^{2}$), but this expression is only valid at equilibrium distance and fails away^40^. A more accurate expression for the hopping parameter is an exponential decay^41^

17 $${t}_{j}={t}_{0}{e}^{-\beta \left(\left|\delta {{^{\prime}}}_{j}\right|/{a}_{0}-1\right)}.$$

where $\beta$ is the Grüneisen parameter. The Grüneisen parameter ($\beta$) is an important thermodynamic quantity that characterizes the behavior of materials under high pressure. In the h-BP case, this parameter has been recently calculated^42^. The Grüneisen parameter definition is

18 $$\beta =-\frac{\partial \mathrm{ln}t}{\partial \mathrm{ln}{a}_{0}}=1.51.$$

### Strain effect on the on-site energy

In addition to the hopping parameters, the on-site energies also can be modified by strain. The linear elastic theory is a method to investigate the strain effects on the on-site energies. Because of the similarity between the graphene and h-BP, we can use results obtained from Ref.^24^. Applying strain changes the total Hamiltonian matrix as follows:

19 $$H=\left(\begin{array}{ll}{\varepsilon }_{p}+\frac{3{a}_{0}}{2}\frac{\partial {\varepsilon }_{p}}{\partial {a}_{0}}\left({\varepsilon }_{xx}+{\varepsilon }_{yy}\right)& \sum_{j=1}^{3}{t}_{j}{e}^{i\overrightarrow{k}.\overrightarrow{{\delta }_{j}}}\\ \sum_{j=1}^{3}{t}_{j}{e}^{-i\overrightarrow{k}.\overrightarrow{{\delta }_{j}}}& {\varepsilon }_{b}+\frac{3{a}_{0}}{2}\frac{\partial {\varepsilon }_{b}}{\partial {a}_{0}}\left({\varepsilon }_{xx}+{\varepsilon }_{yy}\right)\end{array}\right).$$

As mentioned in Ref.^24^, the correction term in the on-site energies is not related to the low-energy Hamiltonian, and we can use it in the full band Hamiltonian, so this justifies the validity of Eq. (19).

The deformation potential matrix is another way to study strain effects on the on-site energies. Applying strain to the h-BP creates a diagonal deformation potential matrix as a function of the Fermi energy and strain. Diagonal potential matrix for the h-BP is^43^

20 $$V=\left(\begin{array}{ll}{g}_{1}\left({\varepsilon }_{xx}+{\varepsilon }_{yy}\right)& 0\\ 0& {g}_{2}\left({\varepsilon }_{xx}+{\varepsilon }_{yy}\right)\end{array}\right),$$

where ${g}_{1}$ and ${g}_{2}$ are a function of the Fermi energy. The existence of two different atoms in the h-BP creates a difference in the diagonal potentials, resulting in ${g}_{1}$ $\ne$ ${g}_{2}$. This is different from graphene, where diagonal potentials are equal to the Fermi energy (${g}_{1}={g}_{2}={E}_{F}$)^43^. Therefore, the total Hamiltonian after applying strain is as follows

21 $$H=H_0+V=\left(\begin{array}{ll}\varepsilon {{^{\prime}}}_{p}& \sum_{j=1}^{3}{t}_{j}{e}^{i\overrightarrow{k}.\overrightarrow{{\delta }_{j}}}\\ \sum_{j=1}^{3}{t}_{j}{e}^{-i\overrightarrow{k}.\overrightarrow{{\delta }_{j}}}& \varepsilon {{^{\prime}}}_{b}\end{array}\right).$$

Here ${t}_{j}$ is the modified hopping parameter by strain. So the new on-site energies after applying strain to the h-BP are $\varepsilon {{^{\prime}}}_{p}={\varepsilon }_{p}+{g}_{1}\left({\varepsilon }_{xx}+{\varepsilon }_{yy}\right)$ and $\varepsilon {{^{\prime}}}_{b}={\varepsilon }_{b}+{g}_{2}\left({\varepsilon }_{xx}+{\varepsilon }_{yy}\right)$. By comparing Eqs. (19) and (21) we can connect the parameters in refs.^24,43^

22 $${g}_{1}=\frac{3{a}_{0}}{2}\frac{\partial {\varepsilon }_{p}}{\partial {a}_{0}}.$$

23 $${g}_{2}=\frac{3{a}_{0}}{2}\frac{\partial {\varepsilon }_{b}}{\partial {a}_{0}}.$$

To calculate parameters ${g}_{1}$ and ${g}_{2}$, we have to compare our results with DFT. For this purpose, we plotted band gap variation versus strains and fit that by results obtained from ref.^44^.

### Electronic density of states

Using Green’s function method, we can calculate the density of states (DOS) of the pristine and strain-induced h-BP. We can obtain the density of states of the h-BP by summing over the FBZ as follows

24 $$D\left(\varepsilon \right)=-\frac{1}{{N}_{c}\pi }\sum_{k}\mathfrak{I}\left[TrG\left(\overrightarrow{k},\varepsilon \right)\right],$$

where ${N}_{c}=2$ denotes the number of atoms per unit cell and non-interacting Green’s function matrix is obtained by $G\left(\overrightarrow{k},\varepsilon \right)={\left[\varepsilon +i\eta -H\left(\overrightarrow{k}\right)\right]}^{-1}$ where $\eta$ is broadening factor. The Green function matrix is

25 $$G\left(\overrightarrow{k},\varepsilon \right)=\left(\begin{array}{cc}{G}_{xx}& {G}_{xy}\\ {G}_{yx}& {G}_{yy}\end{array}\right),$$

Using Eqs. (24) and (25) total DOS reads

26 $$D\left(\varepsilon \right)=-\frac{1}{{N}_{c}\pi }\sum_{k}\mathfrak{I}\left[{G}_{xx}+{G}_{yy}\right].$$

The partial density of states (PDOS) can be calculated straightforwardly as

27 $$PDO{S}_{i}=-\frac{1}{\pi }\mathfrak{I}\sum_{\alpha ,\alpha {^{\prime}}}\langle {O}_{i}|\alpha \rangle {G}_{\alpha \alpha {^{\prime}}}\langle \alpha {^{\prime}}|{O}_{i}\rangle$$

where $i$ is the PDOS index and ${O}_{i}$ represents the eigenvector of the $i$ state.

### Optical conductivity

In general, Ohm’s law is $J=\sigma E$ where $J$ is the current density, $E$ is the electric field, and $\sigma$ is the optical conductivity tensor.

28 $$\sigma =\left(\begin{array}{cc}{\sigma }_{xx}& {\sigma }_{xy}\\ {\sigma }_{yx}& {\sigma }_{yy}\end{array}\right),$$

To calculate the optical conductivity, first, we need the direction-depended velocities. For this purpose, we can write the strain-induced Hamiltonian as

29 $$H=\left(\begin{array}{cc}\varepsilon {{^{\prime}}}_{p}& {g}_{k}\\ {g}_{k}^{*}& \varepsilon {{^{\prime}}}_{b}\end{array}\right),$$

where ${g}_{k}=\sum_{j=1}^{3}{t}_{j}{e}^{i\overrightarrow{k}.\overrightarrow{{\delta }_{j}}}$. The current operator definition is ${j}_{\mu }=e\partial H/\partial {k}_{\mu }$

30 $${j}_{\mu }=e\left(\begin{array}{ll}0& \frac{\partial {g}_{k}}{\partial {k}_{\mu }}\\ \frac{\partial {g}_{k}^{*}}{\partial {k}_{\mu }}& 0\end{array}\right),$$

Also, the general form of the current operator is

31 $$j_{\mu } = - \frac{e}{\hbar }\sum_{k} c_{k}^{\dag } c_{k} \alpha_{k}^{\mu } + i\frac{e}{\hbar }\sum_{k} c_{k}^{\dag } c_{k} \beta_{k}^{\mu }$$

that ${\alpha }_{k}^{\mu }$ and ${\beta }_{k}^{\mu }$ are intra-band and inter-band direction-depended velocities along the $\mu$-direction.

By using linear response theory, the optical conductivity given as

32 $${\sigma }_{\mu \mu {^{\prime}}}\left(\omega \right)=\frac{{g}_{s}}{\mathrm{\hslash }\omega S}\int dt{e}^{i\omega t}\langle \left[{j}_{\mu }\left(t\right),{j}_{\mu {^{\prime}}}\left(0\right)\right]\rangle ,$$

where ${g}_{s}=2$ is the spin degeneracy, $\omega$ is photon frequency, and $S$ is the 2D planar area.

Using Eq. (32), IOC is given as^45,46^

33 $$\begin{array}{cc}{\sigma }_{\mu \mu {^{\prime}}}^{inter}\left(\omega \right)& =i\frac{{g}_{s}{e}^{2}}{{\mathrm{\hslash }}^{2}\omega S}\sum_{k}\left({\beta }_{k}^{\mu }{\beta }_{k}^{\mu {^{\prime}}}\right)[\frac{{f}_{k,c}-{f}_{k,v}}{\mathrm{\hslash }\omega +\Delta E+i{\eta }_{1}}\\ & -\frac{{f}_{k,c}-{f}_{k,v}}{\mathrm{\hslash }\omega -\Delta E+i{\eta }_{1}}].\end{array}$$

where ${f}_{k,c}$, and ${f}_{k,v}$ are Fermi–Dirac distributions in the conduction and valence bands respectively, ${\eta }_{1}$ is the finite damping between the conduction and valence bands, and $\Delta E={E}_{c}-{E}_{v}$ is the energy difference where ${E}_{c}$ and ${E}_{v}$ refer to the conduction and valence bands energies respectively and ${\beta }_{k}^{\mu }=\langle k,c\left|{j}_{\mu }\right|k,v\rangle$ and ${\beta }_{k}^{\mu {^{\prime}}}=\langle k,v\left|{j}_{\mu {^{\prime}}}\right|k,c\rangle$ are velocities along the $\mu$ and $\mu {^{\prime}}$-directions.

Low-frequency photons in the THz region (between the microwave and infra-red) have intra-band optical conductivity that plays the main role in their overall optical conductivity. Intra-band transitions are between a special band and, as shown by Eq. (33), $c=v$ and $\left({f}_{k,c}-{f}_{k,v}\right)/\Delta E$ must be interpreted as $-\partial f\left(k\right)/\partial {\varepsilon }_{j}$. This means that the Drude-like conductivity described by^47^ is responsible for most of the low-frequency photon’s optical behavior.

34 $${\sigma }_{\mu \mu {^{\prime}}}^{intra}=\frac{1}{S}\frac{i{e}^{2}\mathrm{\hslash }}{\mathrm{\hslash }\omega +i{\eta }_{2}}\sum_{kj}{\alpha }_{k}^{\mu }{\alpha }_{k}^{\mu {^{\prime}}}\left(\frac{-\partial f\left(k\right)}{\partial {\varepsilon }_{j}}\right).$$

Here $f\left(k\right)=1/(1+\mathrm{exp}\left(\left({\varepsilon }_{j}-{\mu }_{0}\right)/{k}_{B}T\right))$ is the Fermi–Dirac distribution, ${\varepsilon }_{j}$ is jth eigenvalue, ${\mu }_{0}$ is the chemical potential, ${K}_{B}$ is Boltzmann’s constant, $T$ is temperature, and ${\eta }_{2}$ is the broadening width determined by scattering or disorder in the conduction band. In this paper, we considered $T=$ 10 K.

### EELS of monolayer h-BP

Another important optical property is the EELS. This quantity shows how the energy of scattered host electrons changes by external perturbation. To calculate EELS, we need the dielectric function which is given by

35 $${\varepsilon }_{\mu \mu {^{\prime}}}^{inter}\left(\omega \right)={\varepsilon }_{r}+\frac{i{\sigma }_{\mu \mu {^{\prime}}}^{inter}\left(\omega \right)}{\omega {\varepsilon }_{0}{d}_{BP}},$$

where ${\varepsilon }_{r}$ is the relative permittivity and ${d}_{BP}$ is the h-BP monolayer thickness which we considered 0.4 nm. So the EELS can be calculated as

36 $${L}_{\mu \mu {^{\prime}}}^{inter}=-\mathfrak{I}\left[\frac{1}{{\varepsilon }_{\mu \mu {^{\prime}}}\left(\omega \right)}\right].$$

So we can calculate EELS along the AC and ZZ directions having optical conductivity.

## Discussion

**Figure 4:**
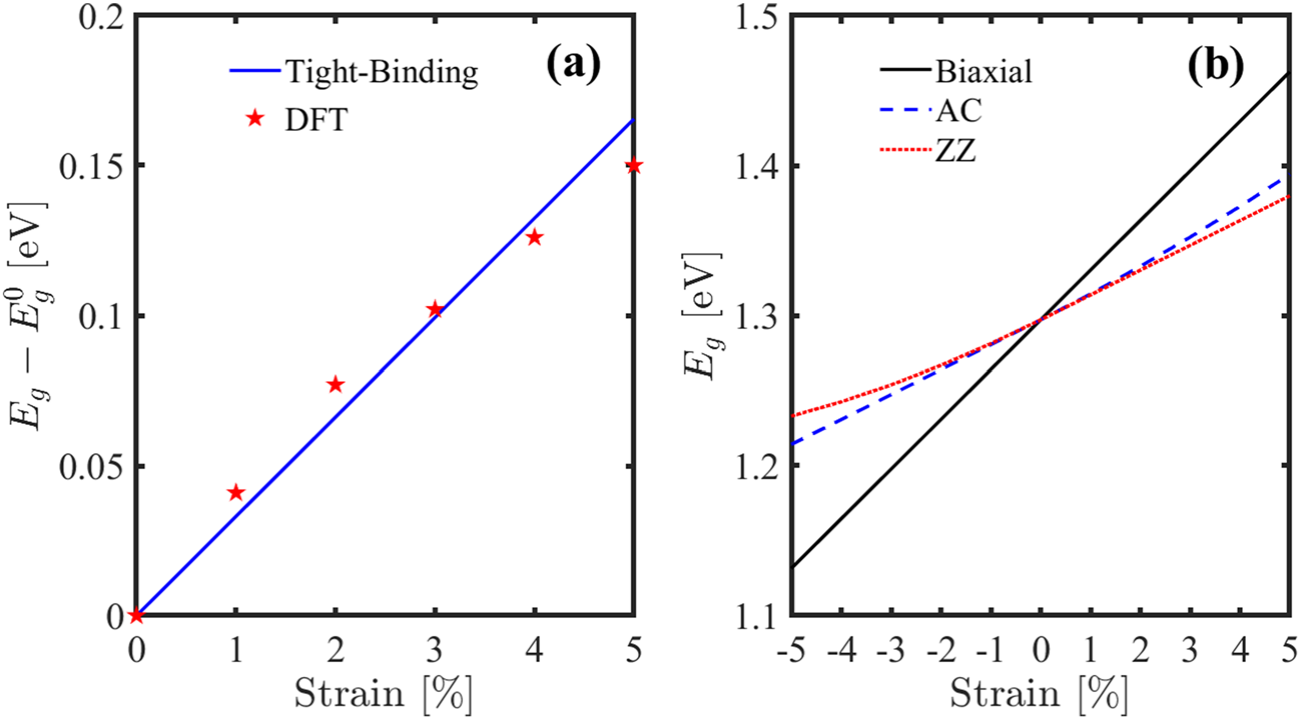
(**a**) Comparison of the band gap variation by strain obtained from tight-binding and DFT calculations^44^. The fitting parameters are ${g}_{1}={E}_{F}$ and ${g}_{2}=0.37{E}_{F}$. (**b**) Band gap versus compressive and tensile strain along the different directions.

Applying strain to the h-BP changes the bond lengths and hopping parameters and displaces the high symmetry points. The band gap is one of the most important properties of the materials which can be modified by strain. Due to the importance of the band gap and for fitting the tight-binding results with DFT calculations and obtaining the parameters ${g}_{1}$ and ${g}_{2}$, we compare the band gap variation plot with the results obtained from a recent DFT study^44^ and obtain the best approximation for ${g}_{1}$ and ${g}_{2}$. Due to the reporting of different band gaps using DFT and tight-binding methods, we compared the gap variation of the two methods by strain to fit the curves. As we can see in Fig. 4a, for ${g}_{1}=-2.6275$ eV and ${g}_{2}=0.37{g}_{1}$ we have a very good match up to 5% stretch. Using these values for ${g}_{1}$ and ${g}_{2}$, we plotted the changes of the band gap for strain along the AC and ZZ directions and biaxial strain (see Fig. 4b). The most effective method to change the gap is to use biaxial strain so that the minimum and maximum values of the gap for compressive and tensile strain are 1.14 and 1.45 eV respectively. But applying strain along the AC and ZZ directions is not as effective as the biaxial strain, and for strain in these directions, the gap changes are almost similar. It’s important to note that applying strain in the discussed ranges does not cause a phase transition in h-BP. Also, our results are in good agreement with the recent first principle studies^44,48^.

**Figure 5:**
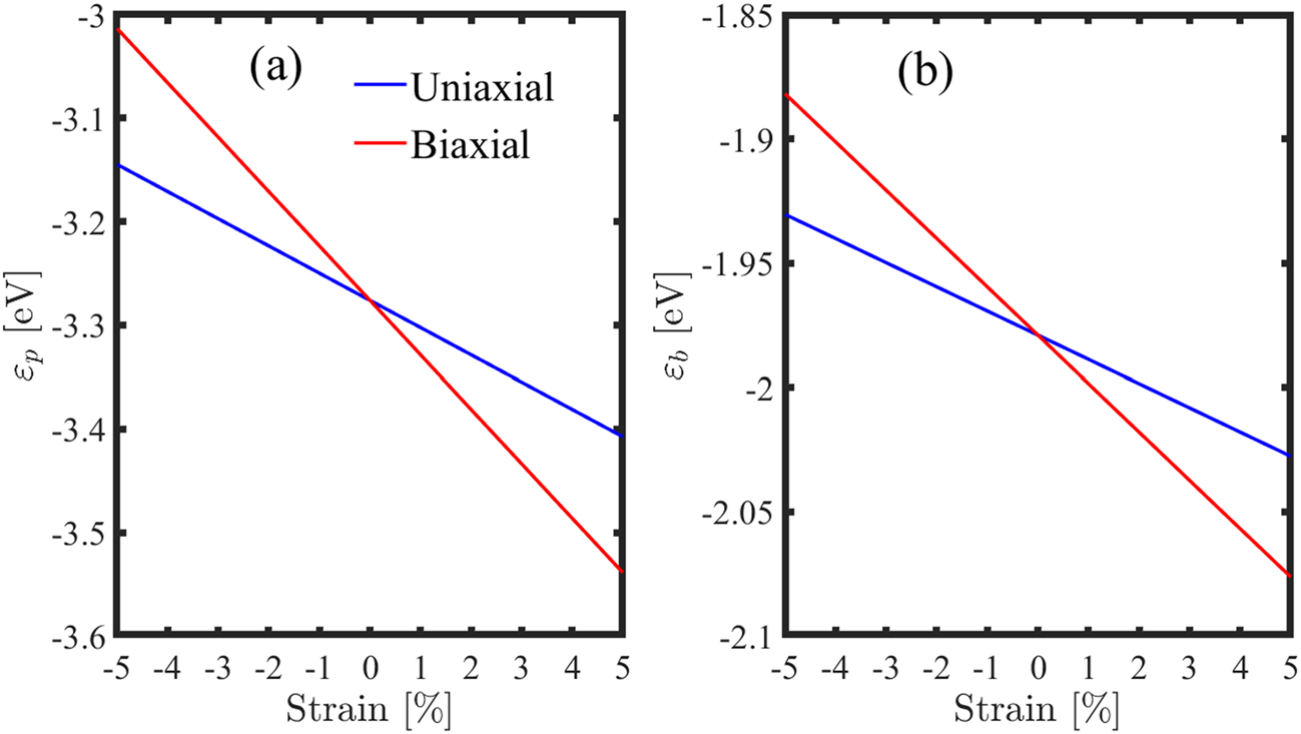
On-site energies versus compressive and tensile strain for (**a**) phosphorus and (**b**) boron atoms.

One particularly interesting effect of strain is its ability to change the energy levels of individual atoms. For example, when a material is strained in one direction, it can cause an increase in the on-site energy of certain boron and phosphorus atoms. This phenomenon is clearly seen in Fig. 5, which shows how on-site energies vary with applied strain. As we can see, tensile strain increases the magnitude of the on-site energies, and compressive strains cause to reduce these parameters.

**Figure 6:**
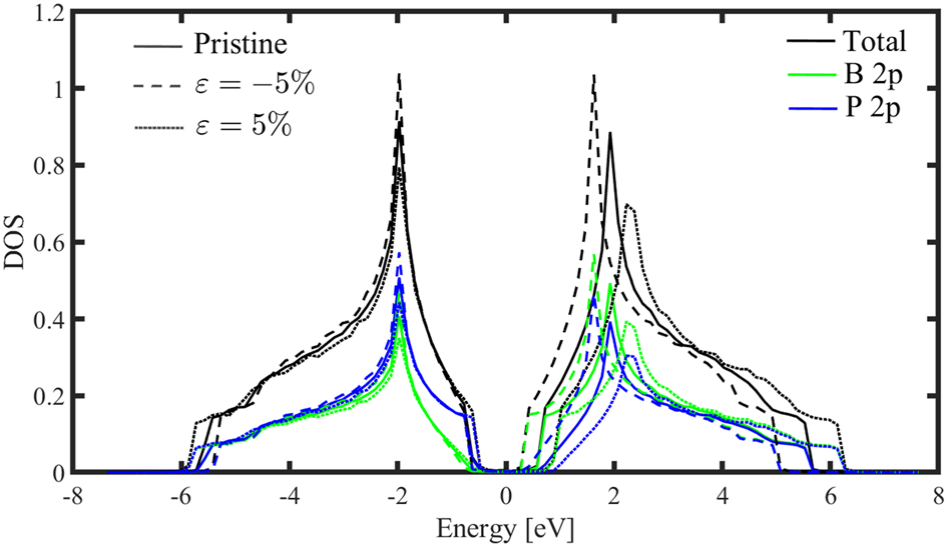
Total and partial density of states of the pristine and strained h-BP. The Fermi level for the pristine case is set at zero.

In addition to the band structure, we can also use the DOS to investigate the strain effects on the electronic phase. We calculated the h-BP partial and total density of states in different strains. Because of the near behavior of the h-BP density of states for applied strains in different directions, we studied only the biaxial strain. From Fig. 6 can be found that the h-BP band gap increases by applying strain. In addition, we can see that the main contribution of two valence band maximum and conduction band minimum comes from the p-orbital of the P and B atoms. Also, our results are in good agreement with the previous study^49^.

By knowing the electronic properties of the h-BP, let’s turn to optical conductivity. We will study strain effects on the inter-band and intra-band optical conductivity for light polarized along the $x$- and $y$-directions. For low-energy photons, the intra-band optical conductivity plays a major role in determining the material’s optical properties. Because the similarity of the intra-band optical conductivity for light polarized along the $x$- and $y$-directions, ${\sigma }_{yy}^{intra}$ is absent in this work. We can say there is no directional dependence on the intra-band electron transport within the h-BP crystals in the pristine case.

**Figure 7:**
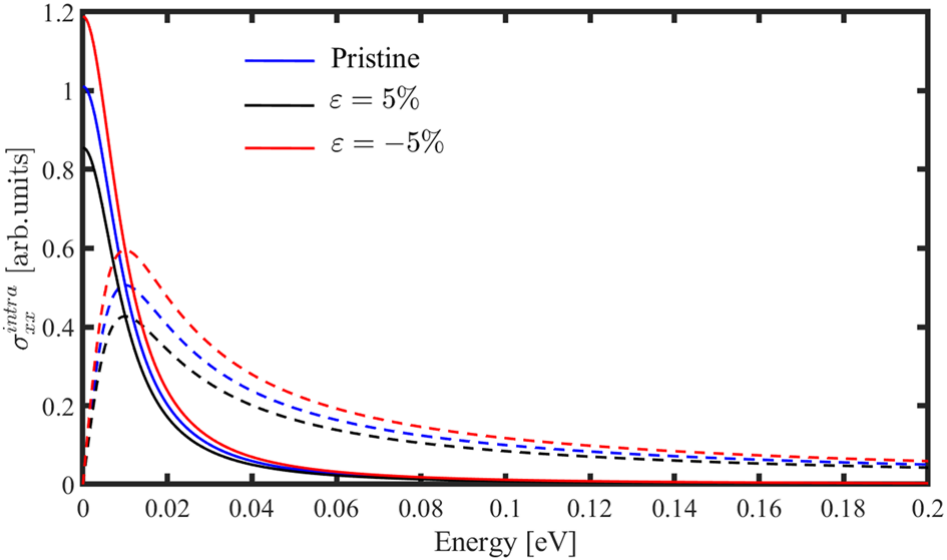
Real (solid lines) and imaginary (dashed lines) parts of the intra-band optical conductivity for light polarized along the x-direction in the presence of biaxial strain by chemical potentials ${\mu }_{c}=0.5 \, {\text{eV}}$ and ${E}_{F}={\mu }_{c}+E(k)$..

Figure 7 shows real and imaginary parts of the intra-band optical conductivity in the presence of the biaxial strain. Our results show a similarity between the intra-band optical conductivity of the h-BP with graphene and ${\beta }_{12}$-borophene^50,51^. Applying strain doesn’t shift the plots and only changes the height of the real and imaginary parts. According to Fig. 7, applying tensile strain up to 5% decreases the intra-band optical conductivity, and compressive strain increases the height of the real and imaginary parts.

**Figure 8:**
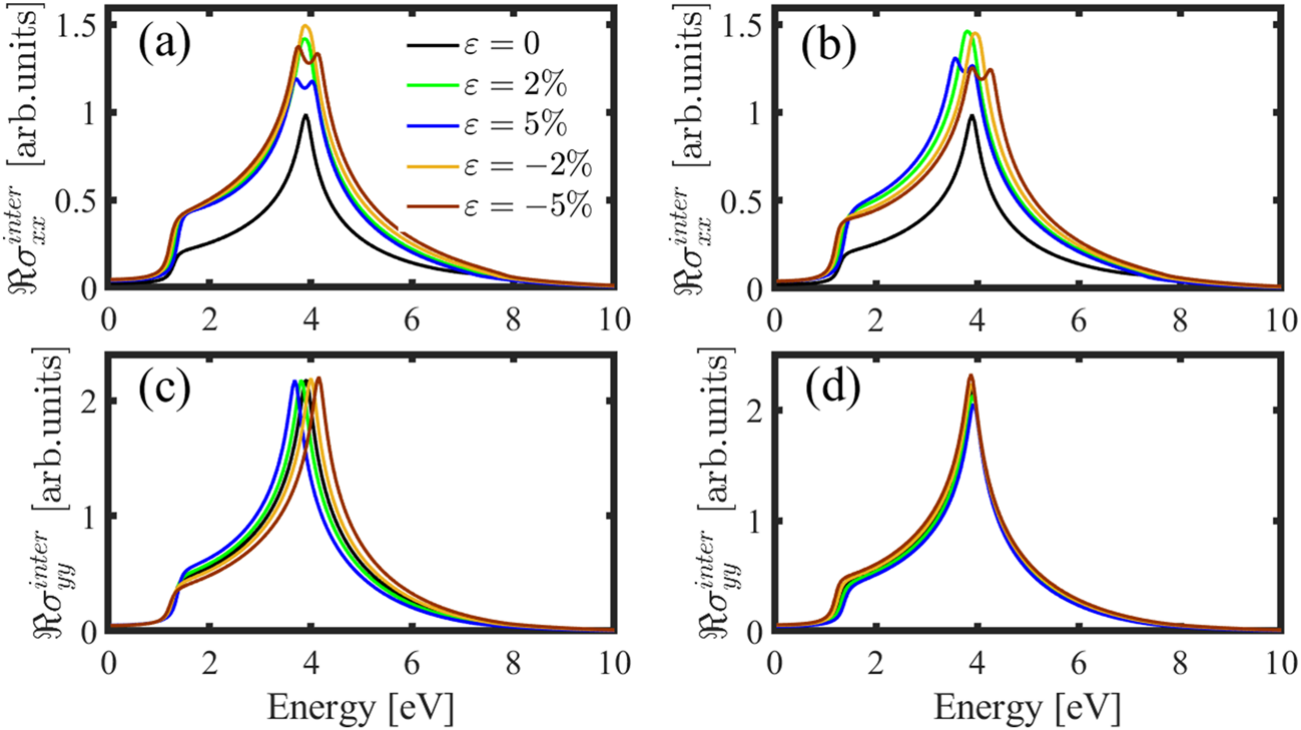
Inter-band optical conductivity for polarized light along the *x*- (**a**, **b**) and *y*-directions (**c**, **d**) in the presence of strain along the AC and ZZ directions respectively.

One particularly promising area of optoelectronics is strain-induced optical modulation. This occurs when an external force is applied to a material that causes it to change shape or size. When this happens, the optical properties of the material also change. If light shines on a strained material, for high-energy photons (higher than THz), the inter-band transitions play a vital role in the optical conductivity. Figure 8a represents the inter-band optical conductivity of the h-BP for light polarized along the $x$-direction under AC strain. In the pristine case, the inter-band optical conductivity behavior is almost similar to that of graphene^52–54^. The absorption peak in the pristine case locates at the energy of about 4 eV, and applying strain along the AC direction causes two peaks to appear around, and a valley at this energy. Also, by applying strain along the AC direction, the height of the optical conductivity is increased. As represented in Fig. 8b, similar to the AC strain, applying strain along the ZZ direction for both tensile and compressive strains (5%) also causes two peaks, but the locations of the peaks are different. Also, for strains less than 5% (compressive and tensile), we only observe an increase in the height of the plots.

The inter-band optical conductivity of the h-BP for polarized light along the $y$-direction (${\sigma }_{yy}^{inter}$) in the pristine case is similar to ${\sigma }_{xx}^{inter}$, so there is isotropic behavior in the optical conductivity of the h-BP in the pristine case, However, uniaxial strain changes this isotropy. Figure 8c shows the real part of the inter-band optical conductivity for light polarized along the $y$-direction under the AC strain. As it’s clear, tensile (compressive) AC strain causes to shift of the absorption peak to the lower (higher) energies, and a redshift (blueshift) occurs. Also, applying strain doesn’t cause two peaks in the optical conductivity in ${\sigma }_{yy}^{inter}$. As we can see in Fig. 8d applying strain along the ZZ direction doesn’t shift the peak’s energy and has a little effect on the height of the plots.

**Figure 9:**
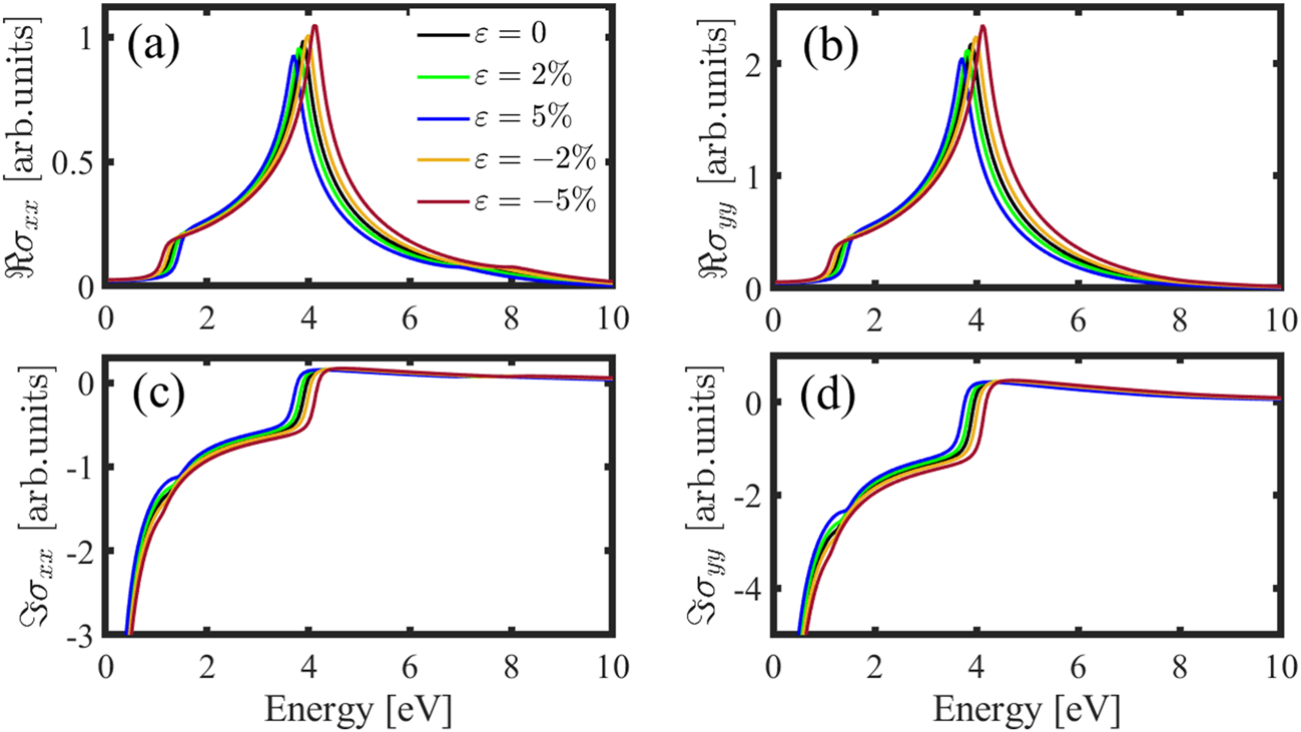
Real and imaginary parts of the inter-band optical conductivity for polarized light along the *x* (**a**, **c**) and the *y*-directions (**b**, **d**) in the presence of biaxial strain.

Unlike the uniaxial strain, applying the biaxial strain preserves the isotropic behavior of the system, and we have similar behavior in the optical conductivity in different directions. Figure 9 shows this isotropy very well. For the real part of the optical conductivity (Fig. 9a,b) applying tensile strain shifts the plots to the lower energies, and compressive strain causes a blueshift. Also, due to the Kramers–Kronig relation, we have the same behavior in the imaginary parts (see Fig. 9c,d).

**Figure 10:**
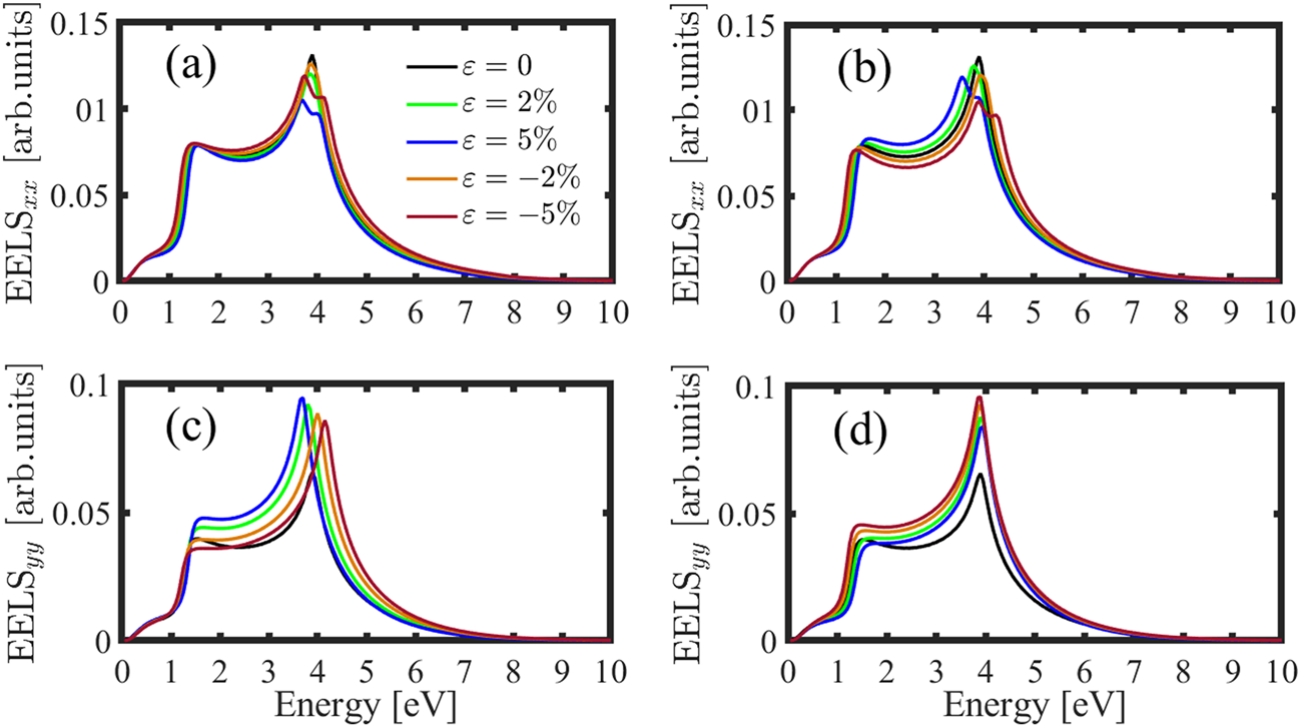
EELS of the perturbed h-BP along the *x* (**a**, **b**) and *y* directions (**c**, **d**) in the presence of the AC and ZZ strain respectively as a function of the photon’s energy.

Another optical quantity is EELS. We plotted this quantity versus the photon’s energy under strain along the AC and ZZ directions (see Fig. 10). This feature behavior is almost similar to the real part of the inter-band optical conductivity. This is because we have most energy lost in the absorption energy. Figure 10a and b shows EELS along the $xx$ direction for strain along the AC and ZZ directions respectively. As we can see two peaks appear for both compressive and tensile strains greater than 2%, This is due to the two absorption peaks in $\mathfrak{R}{\sigma }_{xx}$. EELS of the strained h-BP along the $yy$ direction is different in comparison to the $xx$ direction (see Fig. 10c,d). As it’s clear, for any amount of strain we have only a lost peak almost similar to $\mathfrak{R}{\sigma }_{yy}$. In addition, EELS along the $xx$ direction is greater than the $yy$ and we have more energy lost in this direction.

## Conclusions

In summary, due to the deformation in the structure and orbital hybridization of the atoms, both electronic and optical properties can be modified by strain. The electronic and optical properties of the strained h-BP are depended on the direction of the strain. Applying tensile (compressive) strain in both the AC and ZZ directions increases (decreases) the gap and on-site energies. The maximum and minimum of the band for strain in the range of − 5% to 5% is 1.45 eV and 1.14 eV respectively and are related to the biaxial strain. Our calculation for the DOS confirms the results obtained from the band structure. Our results show the similarity between the intra-band optical conductivity of the pristine h-BP with graphene and ${\beta }_{12}$-borophene. Also, the inter-band optical conductivity of the pristine h-BP is isotropic, but applying uniaxial strain causes anisotropic optical conductivity. Also, we have a similar peak location in the EELS in comparison to the inter-band optical conductivity.

## Data Availability

The data that support the findings of this study are available from the corresponding author upon reasonable request.

## References

[1] Vogt P (2012). Silicene: Compelling experimental evidence for graphenelike two-dimensional silicon. Phys. Rev. Lett..

[2] Li L (2014). Black phosphorus field-effect transistors. Nat. Nanotechnol..

[3] Mannix AJ (2015). Synthesis of borophenes: Anisotropic, two-dimensional boron polymorphs. Science.

[4] Şahin H (2009). Monolayer honeycomb structures of group-IV elements and III–V binary compounds: First-principles calculations. Phys. Rev. B.

[5] Çakır D, Kecik D, Sahin H, Durgun E, Peeters FM (2015). Realization of ap–n junction in a single layer boron-phosphide. Phys. Chem. Chem. Phys..

[6] Zeng B, Li M, Zhang X, Yi Y, Fu L, Long M (2016). First-principles prediction of the electronic structure and carrier mobility in hexagonal boron phosphide sheet and nanoribbons. J. Phys. Chem. C.

[7] Jiang H, Shyy W, Liu M, Wei L, Wu M, Zhao T (2017). Boron phosphide monolayer as a potential anode material for alkali metal-based batteries. J. Mater. Chem. A.

[8] Yu T-T, Gao P-F, Zhang Y, Zhang S-L (2019). Boron-phosphide monolayer as a potential anchoring material for lithium-sulfur batteries: A first-principles study. Appl. Surf. Sci..

[9] Wang S-F, Wu X-J (2015). First-principles study on electronic and optical properties of graphene-like boron phosphide sheets. Chin. J. Chem. Phys..

[10] Kumashiro Y, Nakamura K, Enomoto T, Tanaka M (2011). Preparation and thermoelectric properties of BP films on SOI and sapphire substrates. J. Mater. Sci. Mater. Electron..

[11] Yu J, Guo W (2015). Strain tunable electronic and magnetic properties of pristine and semihydrogenated hexagonal boron phosphide. Appl. Phys. Lett..

[12] Padavala B (2016). Epitaxy of boron phosphide on aluminum nitride (0001)/sapphire substrate. Cryst. Growth Des..

[13] Zhu Z, Cai X, Niu C, Wang C, Jia Y (2016). Computational prediction of the diversity of monolayer boron phosphide allotropes. Appl. Phys. Lett..

[14] Almayyali AOM, Jappor HR (2023). Prediction of new 2D Hf2Br 2N2 monolayer as a promising candidate for photovoltaic applications. Mater. Chem. Phys..

[15] Tareq S, Almayyali AOM, Jappor HR (2022). Prediction of two-dimensional AlBrSe monolayer as a highly efficient photocatalytic for water splitting. Surf. Interfaces.

[16] Bafekry A (2022). Theoretical prediction of two-dimensional BC2X (X = N, P, As) monolayers: ab initio investigations. Sci. Rep..

[17] Chen X (2016). Effect of multilayer structure, stacking order and external electric field on the electrical properties of few-layer boron-phosphide. Phys. Chem. Chem. Phys..

[18] Yarmohammadi M, Mirabbaszadeh K (2020). Electric field tuning of the properties of monolayer hexagonal boron phosphide. J. Appl. Phys..

[19] Wang Y (2019). Stress-and electric-field-induced band gap tuning in hexagonal boron phosphide layers. J. Phys. Condens. Matter.

[20] Mogulkoc Y, Modarresi M, Mogulkoc A, Alkan B (2018). Electronic and optical properties of boron phosphide/blue phosphorus heterostructures. Phys. Chem. Chem. Phys..

[21] Amalia W, Nurwantoro P (2019). Density-functional-theory calculations of structural and electronic properties of vacancies in monolayer hexagonal boron nitride (h-BN). Comput. Condens. Matter.

[22] Chen X (2017). Adsorption of formaldehyde molecule on the pristine and transition metal doped graphene: First-principles study. Appl. Surf. Sci..

[23] Obeid MM (2019). Electronic and magnetic properties of single-layer boron phosphide associated with materials processing defects. Comput. Mater. Sci..

[24] Verberck B, Partoens B, Peeters F, Trauzettel B (2012). Strain-induced band gaps in bilayer graphene. Phys. Rev. B.

[25] Wen Y-N, Xia M-G, Zhang S-L (2018). Bandgap engineering of Janus MoSSe monolayer implemented by Se vacancy. Comput. Mater. Sci..

[26] Winiarski M, Zasada J, Samsel-Czekała M (2018). Strain effects on electronic structures of monolayer iron sulphide and selenide. Comput. Mater. Sci..

[27] Zhang X, Wang S (2021). Interfacial strengthening of graphene/aluminum composites through point defects: A first-principles study. Nanomaterials.

[28] Ni ZH, Yu T, Lu YH, Wang YY, Feng YP, Shen ZX (2008). Uniaxial strain on graphene: Raman spectroscopy study and band-gap opening. ACS Nano.

[29] Han Y (2022). Deep elastic strain engineering of 2D materials and their twisted bilayers. ACS Appl. Mater. Interfaces..

[30] Roldán R, Castellanos-Gomez A, Cappelluti E, Guinea F (2015). Strain engineering in semiconducting two-dimensional crystals. J. Phys. Condens. Matter.

[31] Chun S, Choi Y, Park W (2017). All-graphene strain sensor on soft substrate. Carbon.

[32] Tian H (2014). Scalable fabrication of high-performance and flexible graphene strain sensors. Nanoscale.

[33] Samad YA, Li Y, Alhassan SM, Liao K (2015). Novel graphene foam composite with adjustable sensitivity for sensor applications. ACS Appl. Mater. Interfaces..

[34] Banszerus L (2017). Identifying suitable substrates for high-quality graphene-based heterostructures. 2D Materials.

[35] Gao W, Huang R (2014). Thermomechanics of monolayer graphene: Rippling, thermal expansion and elasticity. J. Mech. Phys. Solids.

[36] Ribeiro R, Pereira VM, Peres N, Briddon P, Neto AC (2009). Strained graphene: Tight-binding and density functional calculations. New J. Phys..

[37] Páez C, Ospina R, Bahamon D (2019). An accurate and compact tight-binding model for GeS. J. Phys. Conf. Ser..

[38] Po HC, Zou L, Senthil T, Vishwanath A (2019). Faithful tight-binding models and fragile topology of magic-angle bilayer graphene. Phys. Rev. B.

[39] Goerbig M (2011). Electronic properties of graphene in a strong magnetic field. Rev. Mod. Phys..

[40] Grosso G, Piermarocchi C (1995). Tight-binding model and interactions scaling laws for silicon and germanium. Phys. Rev. B.

[41] Colombo L (1998). Tight-binding approach to computational materials science. Mater. Res. Soc. Proc..

[42] Bi S (2022). First-principles prediction of the lattice thermal conductivity of two-dimensional (2D) h-BX (X = P, As, Sb) considering the effects of fourth-order and all-order scattering. J. Appl. Phys..

[43] Suzuura H, Ando T (2002). Phonons and electron-phonon scattering in carbon nanotubes. Phys. Rev. B.

[44] Galicia-Hernandez JM, Guerrero-Sanchez J, Ponce-Perez R, Fernandez-Escamilla H, Cocoletzi GH, Takeuchi N (2022). Self-energy corrected band-gap tuning induced by strain in the hexagonal boron phosphide monolayer. Comput. Mater. Sci..

[45] Yang C, Zhang J, Wang G, Zhang C (2018). Dependence of the optical conductivity on the uniaxial and biaxial strains in black phosphorene. Phys. Rev. B.

[46] Yarmohammadi M, Nobahari MM, Tien T, Phuong L (2020). Linear interband optical refraction and absorption in strained black phosphorene. J. Phys. Condens. Matter.

[47] Kupčić I (2009). Incoherent optical conductivity and breakdown of the generalized Drude formula in quasi-one-dimensional bad metallic systems. Phys. Rev. B.

[48] Zhuang HL, Hennig RG (2012). Electronic structures of single-layer boron pnictides. Appl. Phys. Lett..

[49] Khossossi N (2020). Hydrogen storage characteristics of Li and Na decorated 2D boron phosphide. Sustain. Energy Fuels.

[50] Peres N, Ferreira A, Bludov YV, Vasilevskiy M (2012). Light scattering by a medium with a spatially modulated optical conductivity: the case of graphene. J. Phys. Condens. Matter.

[51] Nobahari MM (2022). Anisotropic Kubo conductivity of electric field-induced monolayer β 12-borophene. RSC Adv..

[52] Nguyen VH, Lherbier A, Charlier J-C (2017). Optical Hall effect in strained graphene. 2D Materials.

[53] Pellegrino F, Angilella G, Pucci R (2010). Strain effect on the optical conductivity of graphene. Phys. Rev. B.

[54] Mak KF, Shan J, Heinz TF (2011). Seeing many-body effects in single-and few-layer graphene: Observation of two-dimensional saddle-point excitons. Phys. Rev. Lett..

